# Efficacy and safety of laparoscopic common bile duct exploration via choledochotomy with primary closure for the management of acute cholangitis caused by common bile duct stones

**DOI:** 10.1007/s00464-021-08838-8

**Published:** 2021-11-01

**Authors:** Yanjun Wang, Youbao Huang, Chunfeng Shi, Linpei Wang, Shengwei Liu, Jiawei Zhang, Wei Wang

**Affiliations:** Department of Hepatobiliary Surgery, The Second Affiliated Hospital, Fujian Medical University, 34 Zhongshanbei Road, Quanzhou, 362000 China

**Keywords:** Common bile duct stones, Acute cholangitis, Laparoscopic common bile duct exploration, Primary closure

## Abstract

**Background:**

T-tube drainage after laparoscopic common bile duct exploration (LCBDE) has been demonstrated to be safe and effective for patients with acute cholangitis caused by common bile duct stones (CBDSs). The outcomes after LCBDE with primary closure in patients with CBDS-related acute cholangitis are unknown. The present study aimed to evaluate the efficacy and safety of LCBDE with primary closure for the management of acute cholangitis caused by CBDSs.

**Methods:**

Between June 2015 and June 2020, 368 consecutive patients with choledocholithiasis combined with cholecystolithiasis, who underwent laparoscopic cholecystectomy (LC) + LCBDE in our department, were retrospectively reviewed. A total of 193 patients with CBDS-related acute cholangitis underwent LC + LCBDE with primary closure of the CBD (PC group) and 62 patients underwent LC + LCBDE followed by T-tube placement (T-tube group). A total of 113 patients who did not have cholangitis were excluded. The clinical data were compared and analyzed.

**Results:**

There was no mortality in either group. No significant differences were noted in morbidity, bile leakage rate, retained CBD stones, or readmission rate within 30 days between the two groups. Compared with the T-tube group, the PC group avoided T-tube-related complications and had a shorter operative time (121.12 min vs. 143.37 min) and length of postoperative hospital stay (6.59 days vs. 8.81 days). Moreover, the hospital expenses in the PC group were significantly lower than those in the T-tube group ($4844.47 vs. $5717.22). No biliary stricture occurred during a median follow-up of 18 months in any patient. No significant difference between the two groups was observed in the rate of stone recurrence.

**Conclusions:**

LCBDE with primary closure is a safe and effective treatment for cholangitis caused by CBDSs. LCBDE with primary closure is not inferior to T-tube drainage for the management of CBDS-related acute cholangitis in suitable patients.

Common bile duct stones (CBDSs) are present in approximately 10–15% of individuals with symptomatic gallstones [1]. Because of the biliary obstruction caused by stones, CBDSs often cause serious complications, including acute cholangitis. The current treatments for gallbladder stones with CBDSs mainly include two strategies: endoscopic retrograde cholangiopancreatography (ERCP) + laparoscopic cholecystectomy (LC) and laparoscopic common bile duct exploration (LCBDE) + LC. The optimal approach remains unknown. Although both strategies have shown the same effectiveness and similar complication rates, LCBDE has the advantages of shortened hospitalization duration and lower costs without disruption of the Oddi sphincter [2, 3]. In addition, the ERCP approach may lead to serious complications, including duodenal perforation, pancreatitis and bleeding [4]. As the laparoscopic technique develops and surgeons gain more experience, LCBDE has been gradually used to address CBDS-related cholangitis [5, 6]. Traditionally, LCBDE followed by T-tube placement is a widely adopted method that can drain the bile duct and remove CBD stones [7]. However, T-tube drainage may be associated with T-tube-related complications, such as drain site pain, electrolyte disturbances, biliary retrograde infection and biliary peritonitis due to tube dislodgement, or after T-tube removal [8]. Some of these complications are severe and can require further interventions and thus prolong recovery and increase hospital expenses. With improved methods for intracorporeal suturing and knot tying, LCBDE with primary closure of choledochotomy has become increasingly popular and has been recommended as a possible approach [9, 10]. LCBDE with primary closure is a safe and effective treatment for choledocholithiasis in emergency or elective settings, which has been well documented [11–16]. However, either patients with acute cholangitis were excluded or a small percentage of patients with acute cholangitis were included in these studies, and no specific studies have focused on LCBDE via choledochotomy with primary closure in patients with CBDS-related acute cholangitis. The present study aimed to evaluate the feasibility and safety of LCBDE via choledochotomy with primary closure in patients with CBDS-related acute cholangitis.

## Methods

### Patients

This retrospective clinical study was performed at the Department of Hepatobiliary Surgery of the Second Affiliated Hospital of Fujian Medical University. The protocol of this study was approved by the Institutional Ethics Committee of the Second Affiliated Hospital of Fujian Medical University (No. 19, 2021). Informed consent was obtained from all patients. The data of 368 consecutive patients who were diagnosed with choledocholithiasis combined with cholecystolithiasis and underwent LC + LCBDE via choledochotomy from June 2015 to June 2020 were analyzed retrospectively. Of these 368 patients, 113 patients who did not have cholangitis were excluded. A total of 255 patients with non-severe acute cholangitis (101 males, 154 females; age range, 16–94 years) were divided into two groups according to management patterns: the LC + LCBDE with primary closure group (PC group, *n* = 193) and the LC + LCBDE followed by T-tube placement group (T-tube group, *n* = 62). Stones in the CBD were confirmed by preoperative ultrasonography, magnetic resonance cholangiopancreatography (MRCP), or computed tomography (CT) scans. The diagnosis and severity assessment of acute cholangitis were defined according to the Tokyo Guidelines based on a combination of clinical features, laboratory data and imaging findings [17]. Once a diagnosis was made, the initial treatment, including sufficient fluid replacement, intravenous administration of analgesics and a full dose of antimicrobial agents, was provided. Patients with severe acute cholangitis with organ or system dysfunction were excluded.

### Surgical procedure

**Fig. 1:**
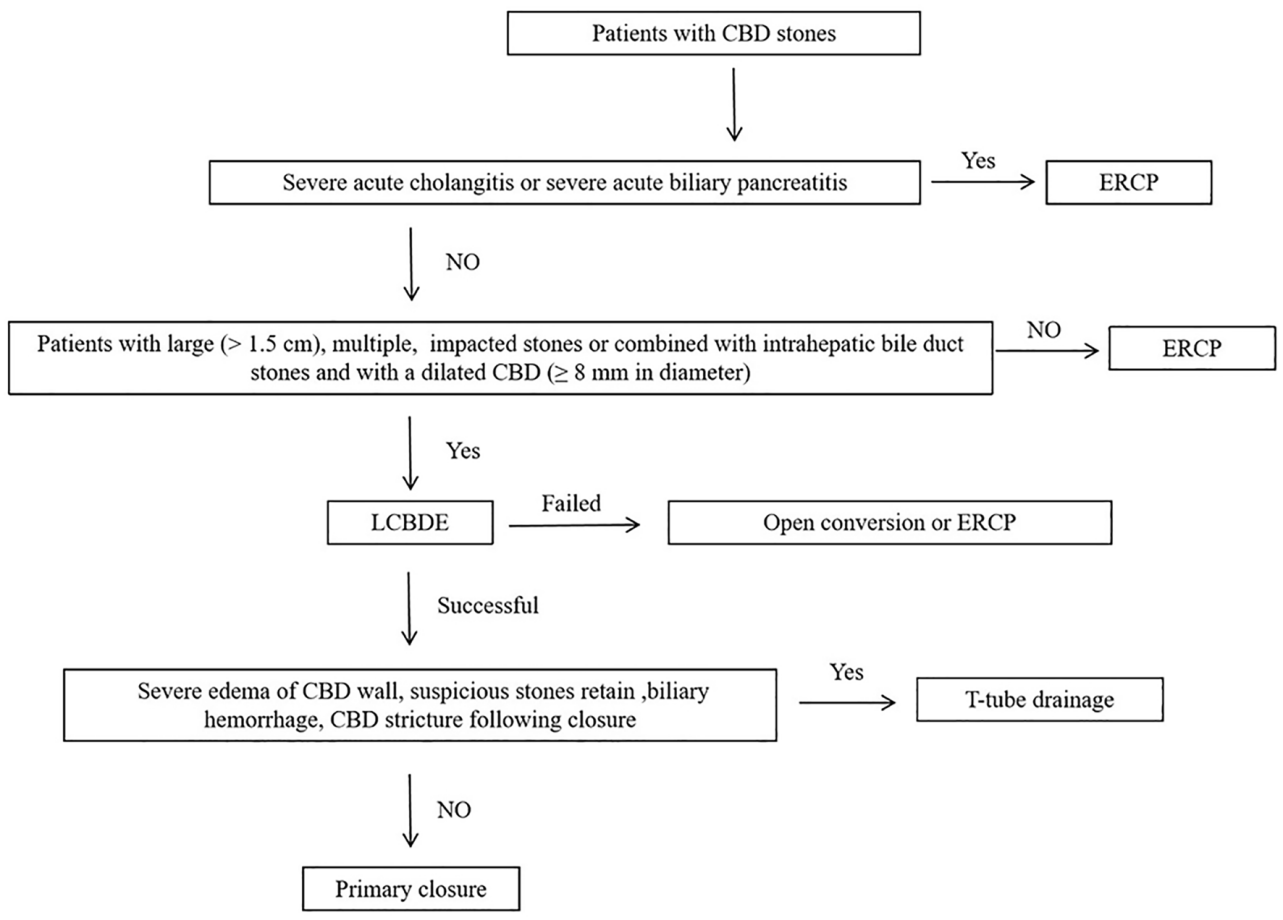
Flow diagram for the management of CBD stones. *CBD* common bile duct, *ERCP* endoscopic retrograde cholangiopancreatography, *LCBDE* laparoscopic common bile duct exploration

LCBDE was performed by experienced laparoscopic biliary surgeons who were experienced in laparoscopic choledochotomy and suturing techniques. The standard four-trocar operative technique was used for LCBDE. Briefly, the operation was started with dissection of Calot’s triangle. The cystic artery was clipped with an absorbable clip and cut off. The cystic duct was clipped and then the gallbladder was dissected free from the liver bed and left behind to facilitate the retraction and exposure of the bile duct during exploration. An incision of approximately 10–15 mm was made along the anterior wall of the CBD longitudinally. A 5-mm flexible choledochoscope (Olympus, Tokyo, Japan) was inserted for CBD exploration. Stones were commonly extracted using saline irrigation or a Dormia basket. If necessary, biliary laser lithotripsy was used to fragment large stones or stones impacted at the ampulla. After complete removal of the stones, choledochoscopy was performed repeatedly to confirm the clearance of the intrahepatic/extrahepatic bile duct and the condition of the distal CBD and Oddi’s sphincter. In the PC group, primary closure of the CBD incision was allowed only if the following conditions were met: (1) CBD stones were confirmed by preoperative MRCP or CT scans with no intrahepatic bile duct stones; (2) the diameter of the CBD was more than 8 mm; (3) no severe edema of the CBD wall was detected intraoperatively; (4) the function of the Oddi sphincter was normal without residual stones; and (5) no biliary hemorrhage was observed. These criteria are similar to previous studies [12, 13, 18, 19]. Then, the incision in the CBD was primarily closed with absorbable 4-0 PDS II (polydioxanone) sutures (Ethicon Inc., Somerville, NJ, USA) in a continuous over-and-over locking fashion. In the T-tube group, an appropriate T-tube was inserted into the CBD incision. The CBD incision was closed using interrupted sutures (4-0 PDS II). Saline was flushed through the T-tube to rule out leakage. A non-suction drain was left in the gallbladder bed, and was removed after 72–96 h if there was no bile leakage postoperatively. The T-tube was removed 6 weeks postoperatively after confirming that no remnant stones or stenosis of the bile duct was present using T-tube cholangiogram and choledochoscopy. A flow diagram for the management of CBD stones in our institution is shown in Fig. 1.

### Data analysis

The demographics, clinical characteristics, and intraoperative, postoperative, and follow-up outcomes were analyzed. The clinical characteristics included age, sex, presentations, laboratory data, severity of acute cholangitis, diameter of the CBD, number of CBD stones, and pathological type of cholecystitis. The morbidity, mortality, duration of surgical procedure, retained stones, length of postoperative hospital stay, and total charges were also compared between the two groups.

### Definitions

The interval between the first incision and closure of the last skin wound was defined as the operative time. The length of postoperative hospital stay was defined as the interval from the day of surgery to the day of discharge. Morbidity and mortality were defined as the number of complications or deaths that occurred in the hospital or within 30 days after surgery. Bile leakage was defined according to the International Study Group of Liver Surgery [20]. Postoperative complications were graded according to the Clavien–Dindo criteria [21]. Every patient was followed up regularly at 3-month intervals. Liver function and radiological examinations were conducted to detect possible bile duct stricture or stone recurrence. The median follow-up time for both groups was 18 months.

### Statistics

Statistical analysis was performed with SPSS 19.0 software (SPSS, Chicago, IL). Continuous data were compared using Student’s *t* test, and categorical data were analyzed by the Chi-square test or Fisher’s exact test. *P* values were two tailed, and *P* < 0.05 was considered significant.

## Results

From June 2015 to June 2020, a total of 255 patients diagnosed with CBDS-related acute cholangitis who underwent LC + LCBDE via choledochotomy were evaluated. The demographic data and clinical characteristics of these patients are presented in Table 1. Two hundred nine patients (81.96%) presented with jaundice and 25 patients (9.80%) presented with the comorbidity of biliary pancreatitis. Moreover, 185 patients (72.55%) presented with multiple CBD stones. The preoperative CBD diameter was 12.31 mm (9.10–23.20) and was ≥ 8 mm in 255 (100%) patients. There was no significant difference in terms of the severity of acute cholangitis, laboratory data, common bile duct diameter, or the pathological type of cholecystitis between the two groups.

**Table 1:** Demographic data and clinical characteristics of patients

	PC group (*n* = 193)	T-tube group (*n* = 62)	*P* value
Age (years)	64 ± 15	66 ± 13	0.322
Sex (male/female)	79/114	24/38	0.756
ASA (I/II/III)	72/102/19	22/35/5	0.855
No. of comorbidity^a^, *n* (%)			
≤ 1	118 (61.14)	41 (66.13)	0.481
≥ 2	75 (38.86)	21 (33.87)	
Presentations, *n* (%)			
Jaundice	161 (83.42)	48 (77.42)	0.285
Right upper quadrant pain	169 (87.56)	51 (82.26)	0.291
Acute biliary pancreatitis	18 (9.33)	7 (11.29)	0.651
Cholangitis (mild)	109 (56.48)	36 (58.06)	0.826
Cholangitis (moderate)	84 (43.52)	26 (41.94)	0.826
Laboratory data			
WBC (× 10^9^/L)	11.12 ± 4.54	11.73 ± 5.12	0.376
ALT (U/L)	181.81 ± 129.50	160.18 ± 157.12	0.279
Total bilirubin (μmol/L)	69.90 ± 47.67	66.67 ± 42.42	0.634
Diameter of CBD (mm)	12.27 ± 2.82	12.44 ± 2.45	0.664
No. of CBD stone, *n* (%)			
Single	55 (28.50)	15 (24.19)	0.509
Multiple	138 (71.50)	47 (75.81)	
Pathologic type of gallbladder, *n* (%)			
Acute cholecystitis	44 (22.80)	15 (24.19)	0.821
Chronic cholecystitis	149 (77.20)	47 (75.81)	

*ASA* American Society of Anesthesiologists, *WBC* white blood cell, *ALT* alanine aminotransferase, *CBD* common bile duct

^a^Arterial hypertension, heart diseases, pulmonary diseases and diabetes mellitus

The intraoperative and postoperative outcomes of the two groups are summarized in Table 2. The operation time was shorter in the PC group than in the T-tube group (121.12 ± 18.24 min vs. 143.37 ± 22.68 min, *P* < 0.01). The postoperative mortality rate was 0% in both groups. There were no statistically significant differences in morbidity, bile leakage rate, retained CBD stones, or readmission rate within 30 days between the two groups (*P* > 0.05), but T-tube-related complications were avoided in the PC group. The T-tube-related complication rate was 11.29% (7/62), which included electrolyte disturbances (3/62), drain site pain (3/62), biliary retrograde infection (1/62), and biliary peritonitis after T-tube removal or accidental displacement (2/62). The length of postoperative hospital stay was shorter in the PC group than in the T-tube group (6.59 ± 1.34 days vs. 8.81 ± 1.85 days, *P* < 0.01). Moreover, the hospital expenses in the PC group were significantly lower than those in the T-tube group ($4844.47 ± $610.44 vs. $5717.22 ± $715.25, *P* < 0.01).

**Table 2:** The intraoperative and postoperative outcomes of the two groups

Outcomes	PC group (*n* = 193)	T-tube group (*n* = 62)	*P* value
Operation time (min)	121.12 ± 18.24	143.37 ± 22.68	0.000
Intraoperative bleeding (mL)	45.65 ± 30.31	38.97 ± 17.75	0.101
Morbidity (*n*, %)	20 (10.36)	10 (15.87)	0.220
Bile leakage (*n*, %)	5 (2.59)	3 (4.84)	0.407
Postoperative bleeding (*n*, %)	3 (1.55)	2 (3.23)	0.598
T-tube-related complication (*n*, %)	0	7 (11.29)	0.000
Electrolyte disturbances (*n*, %)	0	3 (3.84)	
Drain site pain (*n*, %)	0	3 (3.84)	
Biliary retrograde infection (*n*, %)	0	1 (1.61)	
Biliary peritonitis (*n*, %)	0	2 (3.23)	
Pneumonia (*n*, %)	7	2	1.000
Other (*n*, %)	7	2	1.000
Retained CBD stones (*n*, %)	4 (2.07)	3 (4.84)	0.366
Readmission within 30 days (*n*, %)	2 (1.04)	2 (3.23)	0.249
Mortality (*n*, %)	0	0	–
Drain removal (days)	4.53 ± 1.16	4.78 ± 1.35	0.165
Postoperative hospital stay (days)	6.59 ± 1.34	8.81 ± 1.85	0.000
Hospital expenses ($)	4844.47 ± 610.44	5717.22 ± 715.25	0.000

*CBD* common bile duct

The complications and management outcomes are described in Table 3. Eight patients experienced postoperative bile leakage. Of these 8 patients, 4 experienced self-limiting biliary leakage, which was conservatively cured by extended drainage without further procedures. Another three patients had biliary leakage without peritonitis and were treated with extended drainage and intravenous antibiotics. One patient in the PC group experienced subhepatic bile accumulation on postoperative Day 3 and was successfully cured by ultrasound-guided drainage. Nine cases of pulmonary infection were noted in both groups. This condition was cured by treatment with intravenous antibiotics. Postoperative bleeding was observed by peripheral blood testing and/or a fecal occult blood test, and this complication resolved after a 2- to 4-day administration of hemostatic drugs. Seven patients were diagnosed with retained CBD stones. Four patients underwent ERCP to remove the retained CBD stones. With the help of a flexible choledochoscope, the retained CBD stones in three patients were removed through the sinus tract of the T-tube 6 weeks after they underwent LCBDE. The T-tube-related complications of electrolyte disturbances (3/62), drain site pain (3/62), and biliary retrograde infection (1/62) were resolved by conservative treatment. Two patients experienced bile peritonitis (2/62) after T-tube removal or accidental displacement and were cured by ERCP and ultrasound-guided drainage.

**Table 3:** Postoperative complications and management

DC classificationa^a^	Patients	Complications	Management
I	4	Bile leakage	Conservative
II	9	Pulmonary infection	Intravenous antibiotics
	3	Bile leakage	Intravenous antibiotics
	5	Postoperative bleeding	Hemostatic drugs
IIIa	1	Bile leakage	Ultrasound-guided drainage
	3	Retained CBD stone	Postoperative choledochoscopy
IIIb	4	Retained CBD stone	Postoperative ERCP
IV	0	–	–

*DC* Dindo–Clavien, *CBD* common bile duct, *ERCP* endoscopic retrograde cholangiopancreatography

^a^Clavien classification: Grade I: any deviation from the normal postoperative course without the need for pharmacological treatment or surgical, endoscopic and radiological interventions. Grade II: requiring pharmacological treatment with drugs other than such allowed for Grade I complications. Grade III: requiring surgical, endoscopic or radiological interventions; IIIa, intervention not under general anaesthesia; IIIb, intervention under general anaesthesia. Grade IV: life-threatening complications requiring intensive care unit management

The follow-up outcomes are summarized in Table 4. The median time until return to work or full physical activity was shorter in the PC group than in the T-tube group (11.45 ± 4.34 days vs. 19.12 ± 6.34 days, *P* < 0.01). No biliary stricture occurred after LCBDE in any patient, with a median follow-up of 18 (range 6–32 months) months in any patient. No significant difference between the two groups was observed in the rate of stone recurrence (*P* > 0.05). A total of 13 patients with stone recurrence were observed; 9 patients were cured by ERCP, and the other patients underwent reoperation.

**Table 4:** Outcomes following LCBDE

Outcomes	PC group (*n* = 193)	T-tube group (*n* = 62)	*P* value
Time to return to work or full physical activity (days)	11.45 ± 4.34	19.12 ± 6.34	0.000
Bile duct stricture, *n* (%)	0	0	–
Stone recurrence, *n* (%)	9 (4.66)	4 (6.45)	0.524
Management of stone recurrence			
ERCP extraction	7	2	
Reoperation	2	2	

*LCBDE* laparoscopic common bile duct exploration, *ERCP* endoscopic retrograde cholangiopancreatography

## Discussion

In the Tokyo Guidelines 2018 (TG18), endoscopic drainage as the first-line drainage procedure is the recommended treatment for acute cholangitis, regardless of the degree of severity, especially for patients with severe acute cholangitis, except in some cases of mild acute cholangitis in which antibiotics and general supportive care are effective [22]. Previously, two-stage treatment had been recommended for acute cholangitis with bile duct stones, which involves biliary drainage as an initial treatment and endoscopic stone removal after the improvement of cholangitis [23]. Recently, some studies have demonstrated that bile duct stone removal following EST in a single-stage procedure may be considered in patients with mild or moderate acute cholangitis [24, 25]. However, the updated TG18 recommends bile duct stone removal in a two-stage procedure after drainage in patients with a large stone or multiple stones if endoscopic papillary large balloon dilation (EPLBD) is required. Some studies have demonstrated the subset of patients with large (> 1.5 cm), multiple, or impacted stones, which might be difficult to extract with the ERCP approach in a single-stage procedure [26–28]. In addition, our experience with difficult biliary stones (large, multiple, or impacted stones) extracted with the ERCP approach in a single-stage procedure for the management of acute cholangitis is not extensive enough.

With the rapid advancement of minimally invasive surgical techniques, LCBDE has become a widely accepted surgical approach for patients with gallbladder and CBD stones [29]. LCBDE is superior to the ERCP approach in the treatment of choledocholithiasis because it is a single-stage procedure and because it maintains the integrity of the sphincter of Oddi, which is important in young patients [2, 30]. In recent years, accumulating data have demonstrated that LCBDE is safe and feasible for mild or moderate acute cholangitis, but not severe acute cholangitis, due to the high mortality rate [5, 6, 11, 31, 32]. Stone removal at a single session can shorten the hospital stay, and the LCBDE approach is usually performed. Thus, in our center, in the subset of patients with large (> 1.5 cm), multiple, or impacted stones, the LCBDE approach was usually performed by our experienced laparoscopic biliary surgeons. In our current study, 185 patients (72.55%) presented with multiple CBD stones. Ultimately, our choice to perform either ERCP or LCBDE was mostly based on our clinical experience, available equipment, characteristics of the biliary tree, and patient selection, which is consistent with Society of American Gastrointestinal and Endoscopic Surgeons (SAGES) Guidelines and clinical spotlight review for the clinical application of laparoscopic common bile duct exploration [29, 33].

Traditionally, LCBDE is accompanied by the placement of a T-tube [33], which is a traditional practice accepted by several generations of surgeons. However, this practice is associated with significant T-tube-related complications, including drain site pain, persistent biliary fistula, electrolyte disturbances, biliary peritonitis, and an extended period of having the tube before its removal, which is an inconvenience to the patient [34]. The T-tube-related complication rate is approximately 15% [8]. These complications may prolong the hospital stay, delay postoperative recovery and increase medical expenditure. In our present study, the T-tube-related complication rate was 11.29% (7/62) in the T-tube group, which included electrolyte disturbances (3/62), drain site pain (3/62), biliary retrograde infection (1/62), and biliary peritonitis after T-tube removal or accidental displacement (2/62). Furthermore, T-tube insertion after LCBDE was associated with a prolonged hospital stay, a longer operating time, and higher hospital expenses, which is consistent with previous studies [7, 9, 10, 35, 36].

However, at present, the safety and efficacy of LCBDE with primary closure for patients with CBDS-related acute cholangitis are rarely reported in the literature. To the best of our knowledge, this is the largest series in the literature to date of LCBDE via choledochotomy with primary closure for the treatment of CBDS-related acute cholangitis. In this study, there was no significant difference between the two groups in postoperative mortality, postoperative bile leakage, retained stones, recurrence of stones and biliary stricture, or readmission within 30 days.

LCBDE can be performed either through the cystic duct or through a choledochotomy. Recently, some studies have shown high success rates with the transcystic approach [37, 38]. However, the transcystic approach is limited by the location, size, number of stones and diameter of the cystic duct [29]. In our series, most patients presented with multiple, large CBD stones. In addition, our experience with LCBDE through the cystic duct is limited, but the anterior incision of the CBD is a skilled operation, which can reduce the operation time and the risk of the procedure during the acute cholangitis stage. Thus, our surgeons performed LCBDE via choledochotomy with primary closure for the treatment of acute cholangitis caused by common bile duct stones.

Bile leakage negatively influences postoperative recovery and patients might need further interventions or even reoperation. Bile leakage following primary closure is a major criterion for assessing the safety of LCBDE with primary closure for the treatment of CBDS-related cholangitis. In our series, 5 patients (5/193, 2.59%) in the PC group and 3 patients (3/62, 4.84%) in the T-tube group experienced postoperative bile leakage, which is comparable with the bile leakage rates of 2.5–7% reported by other studies [12, 15, 39, 40]. Previous studies have shown that T-tube do not prevent bile leakage [9, 35]. In our study, the presence of the T-tube did not prevent bile leakage, as 3 patients experienced bile leakage when the tube was still in situ. Some studies [14, 15, 18] have shown that surgeon experience and CBD diameter are two important risk factors for bile leakage after primary closure. In our study, LCBDE was performed by surgeons with more than 10 years of experience in hepatobiliary and laparoscopic surgery, and the criteria for primary closure after LCBDE were powerfully executed. In our institution, the indications for primary closure of the CBD incision are as follows: (1) CBD stones with no intrahepatic bile duct stones; (2) CBD diameter greater than 8 mm; (3) no severe edema of the CBD wall; (4) normal function of the Oddi sphincter without residual stones; and (5) no biliary hemorrhage. These criteria are similar to those in previous studies [12, 13, 18, 19].

Retained stones are one of the major complications following LCBDE and have been reported in up to 8.5% of cases [41]. With the application of choledochoscopy and laser lithotripsy equipment, the rate of retained stones has been significantly reduced to approximately 2% [19, 42]. In our series, the rate of retained stones was 2.75% (7/255). This may be due to the use of the choledochoscope and repeated confirmation of duct clearance before duct closure. Retained stones were detected in four patients who underwent LCBDE with primary closure, and the retained stones were removed by ERCP with no additional surgery required.

The recurrence of stones and biliary stricture after LCBDE are rare complications, and a previous long-term study showed that patients with primary closure have low rates of stone recurrence and biliary stricture [43]. In our series, no biliary stricture occurred after LCBDE during a median follow-up of 18 months, which may be due to the strict indications that required patients who underwent LCBDE to have a dilated CBD (≥ 8 mm in diameter). In our study, there was no significant difference between the two groups in stone recurrence rate.

In our series, patients in the PC group who underwent primary duct closure had a significantly shorter operative time and postoperative hospital stay and lower medical expenditure than those who underwent T-tube drainage (*P* < 0.01). Moreover, the median time until return to work or full physical activity was shorter in the PC group than in the T-tube group (*P* < 0.01). These results are comparable with those of previous studies [9, 36, 44]. LCBDE with primary closure in patients accelerates hospital discharge and recovery and lessens hospital expenses. In this new era of laparoscopic surgery, therapeutic methods tend to be increasingly minimally invasive, and LCBDE with primary closure is consistent with the concept of reducing trauma, hastening the patient’s recovery, and reducing the need for hospitalization.

Our current study was a retrospective analysis, and all retrospective analyses may have selection bias. Although the two groups did not have significant differences in the demographic data and clinical characteristics of the patients and the comparison of the two groups in terms of morbidity, mortality, operation time, postoperative hospital stay, and hospital expenses was a fair comparison, and the choice to perform either primary closure or T-tube drainage was, to a certain extent, based on our clinical experience. The aim of the present study was to obtain preliminary data to compare primary closure after LCBDE with conventional T-tube drainage, and the non-inferiority of LCBDE with primary closure is an important finding. Large well designed and adequately powered RCTs that compare primary duct closure and T-tube insertion are still required to validate these observations.

In conclusion, LCBDE with primary duct closure is a safe and effective approach for the management of CBDS-related cholangitis, is not inferior to T-tube drainage and has a shorter operative time, and leads to shorter postoperative hospital stays and lower medical expenditures for patients. A randomized clinical trial is needed to further evaluate the benefits of LCBDE with primary closure in this subgroup.
